# An investigation into DNA methylation patterns associated with risk preference in older individuals

**DOI:** 10.1080/15592294.2021.1992910

**Published:** 2021-10-30

**Authors:** Laura J. Smyth, Sharon M. Cruise, Jianjun Tang, Ian Young, Bernadette McGuinness, Frank Kee, Amy Jayne McKnight

**Affiliations:** a Epidemiology and Public Health Research Group, Centre for Public Health, Queen’s University Belfast, Northern Ireland, UK; b School of Agricultural Economics and Rural Development, Renmin University of China, Beijing, China

**Keywords:** Age, methylation, epigenetics, genes, risk-preference

## Abstract

Risk preference is a complex trait governed by psycho-social, environmental and genetic determinants. We aimed to examine how an individual’s risk preference associates with their epigenetic profile.

Risk preferences were ascertained by asking participants of the Northern Ireland COhort for the Longitudinal study of Ageing to make a series of choices between hypothetical income scenarios. From these, four risk preference categories were derived, ranging from risk-averse to risk-seeking. Illumina’s Infinium High-Density Methylation Assay was used to evaluate the status of 862,927 CpGs.

Risk preference and DNA methylation data were obtained for 1,656 individuals. The distribution of single-site DNA methylation levels between risk-averse and risk-seeking individuals was assessed whilst adjusting for age, sex and peripheral white cell counts. In this discovery cohort, 55 CpGs were identified with significantly different levels of methylation (p≤x10^−5^) between risk-averse and risk-seeking individuals when adjusting for the maximum number of covariates. No CpGs were significantly differentially methylated in any of the risk preference groups at an epigenome-wide association level (p<9x10^−8^) following covariate adjustment.

Protein-coding genes *NWD1* and *LRP1* were among the genes in which the top-ranked dmCpGs were located for all analyses conducted. Mutations in these genes have previously been linked to neurological conditions.

Epigenetic modifications have not previously been linked to risk-aversion using a population cohort, but may represent important biomarkers of accumulated, complex determinants of this trait. Several striking results from this study support further analysis of DNA methylation as an important link between measurable biomarkers and health behaviours.

## Introduction

There is mounting evidence to link social factors and genetic variance. Thus, the social environment can affect gene expression and an individual’s genotype can alter their sensitivity to the social environment [1]. Individual-level genetic and epigenetic associations with a wide variety of social science phenotypes including, intelligence [2], susceptibility to post-traumatic stress disorder (PTSD) [3], socioeconomic status (SES) [4], subjective well-being [5] and both economic and political preferences [6] have been evaluated.

Risk-preference, our willingness to take risks and decision-making under uncertainty, involves a trade-off between small high-probability rewards and large low-probability rewards and has been thoroughly investigated in behavioural economics, neuro-economics and psychology. Risk preference is thought to be affected by an individual’s biological, demographic, socioeconomic and psychological status [7]. Individual risk perceptions have been assessed in conjunction with several health behaviours and traits, where it has been shown that individuals who are more risk-tolerant have an increased likelihood of engaging in behaviours that present risks to their health such as heavy alcohol consumption [8] and smoking [9].

Gene–environment interactions are thought to mediate the emergence of complex behavioural phenotypes [1,10]. An understanding of both social and genetic risks across the life-course is crucial to identify pathways determining health behaviours and health outcomes [11]. Given the broad associations between risk preference and a variety of behaviours affecting public health, it is important to better understand both their genetic and social origins. Evidence suggests that both individual genotypes and environmental influences have been associated with, and are predictive of economic risk preferences [6].

Epigenetics refers to heritable and dynamic alterations that complement a person’s inherited nucleotide sequence; these often lead to changes in gene expression [12]. Emerging evidence has indicated that various epigenetic alterations, both those to which the individual is predisposed and those which they acquire throughout life, are key factors in the development of several complex diseases [12]. Epigenetic modifications may serve as critical mechanisms through which social exposures occurring over the life-course can have sustained effects on behaviour and psychological traits [13–15]. Epigenetic alterations differ from genetic mutations in that the changes can be independent of the inherited nucleotide coding sequence, reversible and induced by both drugs and environmental stimuli [13]. DNA methylation is a common, stable epigenetic modification, with the ability to affect gene expression by altering the binding patterns of transcriptional proteins [16].

Increased incorporation of genetic and epigenetic data into longitudinal cohorts and social science surveys has provided novel opportunities to correlate complex genetic-epigenetic variants to observed social and economic traits of relevance to public health [17].

In this study, we examined the associations between the epigenetic profiles and risk preferences of a sample of community dwelling older adults. Financial decisions and health behaviours were examined using newly available data from the Northern Ireland COhort for the Longitudinal study of Ageing (NICOLA, https://www.qub.ac.uk/sites/NICOLA/AboutNICOLA/). The aim of this investigation was to examine if an individual’s risk preference associates with their DNA methylation epigenetic profile.

## Material and methods

### NICOLA

The NICOLA project is the first large-scale longitudinal study of ageing in Northern Ireland, created to gain a better understanding of the factors affecting social and health outcomes in the older Northern Irish population and has been designed to maximize comparability with other established international longitudinal studies including the Health and Retirement Study in the United States. In wave 1, NICOLA recruited a random sample of 8,504 individuals (a 63% response rate), 8,309 of whom were aged 50 years or older. Spouses or partners of participants who shared their addresses were also invited to participate, regardless of age. Recruited individuals were asked to complete a computer-assisted personal interview (CAPI) and self-completion questionnaire (SCQ) to capture additional information, completed by 8,478 and 5,032 individuals, respectively, [18].

### Determination of risk preferences

In order to ascertain their risk preference, a randomly selected group of 4,564 individuals included in the NICOLA study were asked to make a series of choices between two hypothetical income scenarios as part of their SCQ [19]. The series of questions were as follows:

*Imagine the following hypothetical situations. For each of these three choices below, which income do you choose?*

#### Choice 1

***1A*** *Income A, which will with certainty give you a £1,500 per month for the rest of your life.*

***1B*** *Income B, which will give you a 50–50 chance of £3,000 and a 50–50 chance of £1,000 per month for the rest of your life.*

#### Choice 2

***2A*** *Income A, which will with certainty give you a £1,500 per month for the rest of your life.*

***2B*** *Income B, which will give you a 50–50 chance of £3,000 and a 50–50 chance of £1,200 per month for the rest of your life.*

#### Choice 3

***3A*** *Income A, which will with certainty give you a £1,500 per month for the rest of your life.*

***3B*** *Income B, which will give you a 50–50 chance of £3,000 and a 50–50 chance of £1,300 per month for the rest of your life.*

Offer A offers a sure amount, in contrast to option B which offers a chance to receive either higher or lower amounts. Risk-averting participants would choose option A and a higher number of option A chosen implies a higher risk-averse nature. Based on participants’ choices to the three income scenarios described above, participants were categorized into one of four mutually exclusive groups to create a ‘risk preference’ derived variable, as follows: risk-averse (chose 1A, 2A, and 3A); mildly risk-averse (chose 1A, 2A, and 3B); mildly risk-seeking (chose A1, 2B, and 3B); and risk-seeking (chose 1B, 2B, and 3B). Participants selecting alternative combinations of options to those described were excluded from the analysis [20]. Depending on their responses, the individuals were allocated to one of four groups, ranging from individuals who were shown to be more risk-averse to those more likely to take risks. These groups were labelled risk-averse, mildly risk-averse, mildly risk-seeking, and risk-seeking.

### Laboratory methodology

Blood samples were collected from 1,980 NICOLA participants in EDTA tubes. Samples were processed by Eurofins Scientific, who extracted the DNA from buffy coats and performed quantitation using PicoGreen.

The EZ Zymo Methylation Kit (Zymo Research, USA) was used to bisulphite treat the DNA following the alternative overnight incubation conditions provided in the published protocol for use with the Illumina® Infinium MethylationEPIC Kit. All samples were prepared and analysed using the Infinium MethylationEPIC Kit and BeadChips (Illumina, USA) with no protocol deviations. Participant samples were randomly distributed across 249 arrays. Duplicates of eight samples were located on a separate array to their paired sample. The array on which the duplicate samples were run was randomly allocated a position mid-way through all arrays processed for this study. Due to the positioning of the samples in the arrays, we were unable to determine within-array concordance for duplicate samples. DNA obtained from each individual was treated in a consistent manner, with standard quality control (QC) applied which included evaluation of the bisulphite treatment conversion efficiency, dye specificity, hybridization and staining. This was assessed using GenomeStudio v2011 and BeadArray Controls Reporter software platforms using a pre-set standard set of controls (both Illumina). All participants included in this analysis were White Caucasian.

### QC and data analysis

Proportional white cell counts (WCCs) were estimated using the Houseman method [21], the *minfi* Bioconductor (v3.10) package and the raw .idat files which were output from the iScan machine. Estimation of six WCCs was performed using the *estimateCellCounts* function. QC, pre-processing and differential methylation analyses were undertaken in the R statistical environment (3.6.3) utilising RnBeads and Bioconductor packages. Cross-reactive probes and those located within three base pairs of common SNPs were excluded due to their abilities to map to multiple areas of the genome and affect probe hybridization respectively. Unreliable probes and samples were removed by RnBeads initially by the Greedycut algorithm (p<0.05). Those located on sex chromosomes were also removed. Raw intensities were normalized using the bmiq method.

All software was used following the developer’s instructions. Beta values were generated and M values were derived for all sites. P-values were computed using the limma method for each site. Hierarchical linear models from the limma package were employed and fitted using a Bayesian approach on the derived M values. P-values were generated for each of the four differential analyses conducted.

Four main analyses were performed using RnBeads. In each analysis, the dmCpGs were identified and marked differences (p≤x10^−5^) were reported with CpG locations mapped to Human Genome build 37. Only individuals who had been randomly allocated to the hypothetical income scenario question group and had provided answers, and had both donated blood samples and consented to methylation analyses, were included.

**Figure 1.**
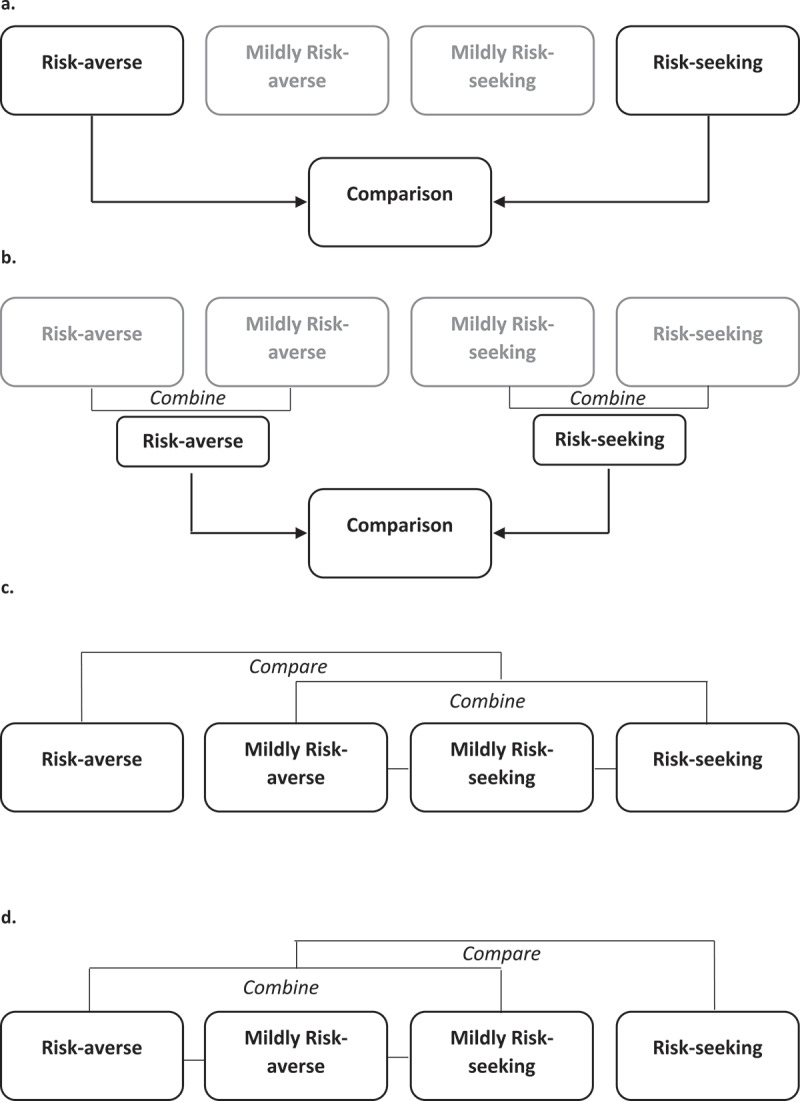
Analysis outlines.
- *Analysis 2: Assessment of single-site methylation levels in all risk-averse vs. all risk-seeking individuals. A*ll *risk-averse* (*risk-averse* and *mildly risk-averse*) individuals were compared all *risk-seeking* (*risk-seeking* and *mildly risk-seeking)* individuals, Figure 1(b).
- *Analysis 3: Assessment of DNA methylation in the risk-averse group, compared to the remaining population* (Figure 1(c)).
- *Analysis 4: Assessment of DNA methylation in the risk seeking group, compared to the remaining population* (Figure 1(d)).

- *Analysis 1: Assessment of single-site methylation levels in risk-averse vs. risk-seeking individuals*. Top-ranked dmCpGs were identified following the comparison of *risk-averse* and *risk-seeking* population groups (Figure 1(a)).

The mildly risk-averse and mildly risk-seeking population groups were not included in Analysis 1 to increase the discrimination between the risk-averse and risk-seeking population groups. These population groups have been included in Analyses 2, 3, and 4.

Two adjustment models were run for each analysis. Age, sex and the six WCCs were adjusted for in the minimal model. The maximal model included age, sex, six WCCs, whether the individual was taking any mental health medication, deprivation level, marital status, education level, smoking status, alcohol intake and BMI.

A methylation p-value threshold of p<9x10^−8^ is considered to adequately control the false-positive rate for DNA methylation data obtained using the Infinium MethylationEPIC array [22]. A suggested significance level of p≤x10^−5^ [23] was utilized in this discovery cohort to identify those dmCpGs, which may be linked to risk preferences and are ideally suited for further analysis. Supporting data for dmCpGs were sought from published literature and *in silico* functional analyses.

Using pwrEWAS [24], we have >77% power to detect a true association defined as detected dmCpGs using this cohort of individuals with a 10% difference in CpG methylation levels (P_FDRad_j≤0.05), and >88% power to detect a 20% difference in CpG methylation levels (P_FDRad_j≤0.05) when comparing the *risk-averse* cohort of individuals to the remaining population and the *risk-seeking* cohort of individuals to the remaining population.

### Functional network analyses

Top-ranked dmCpG containing genes arising from both analysis models were processed using gene ontology and pathway enrichment analysis (p≤x10^−5^) using the Database for Annotation, Visualization, and Integrated Discovery (DAVID) web tool [25].

## Results

### NICOLA participant data

From the 8,452 NICOLA participants included in *Wave 1* of the social survey data collection, risk preference data was collected for 4,582 individuals. Of these participants, blood samples for methylation analysis were collected for 1,656 individuals. Table 1 shows the breakdown of NICOLA participants with risk preference data, with and without methylation data. A chi-square test was performed to ensure that the distribution of risk preferences in the population of individuals with both methylation data and risk preference data was not different to the population who did not consent to methylation analysis. A p-value of 0.014 was ascertained, demonstrating that the distribution of risk preferences of the two populations was not significantly different at the recommended 1% level [26]. Following the coding of the answers provided as part of the SCQ, the participants were allocated to four risk preference groups, ranging from *risk-averse* to *risk-seeking*. This breakdown is shown in Table 2, alongside the data pertaining to the additional covariates.

**Table 1.** NICOLA population data demonstrating the breakdown of participants with risk preference data within NICOLA both with and without Infinium MethylationEPIC data.

Population	Risk averse	Mildly risk averse	Mildly risk seeking	Risk seeking	Total
**Individuals with risk preference data who consented to methylation analysis (%)**	1,034 (62.4)	260 (15.7)	183 (11.1)	179 (10.8)	1,656
**Individuals with risk preference data who did not consent to methylation analysis (%)**	1,963 (67.1)	401 (13.7)	297 (10.2)	265 (9.1)	2,926
**All individuals with risk preference data from NICOLA (%)**	2,997 (65.4)	661 (14.4)	480 (10.5)	444 (9.7)	4,582

**Table 2.** Breakdown of risk preference groups and associated covariates.

		Population per category					
Variable	Population	Risk-averse *(%)*	Mildly risk-averse *(%)*	Mildly risk-seeking *(%)*	Risk-seeking *(%)*	Total	*P* value (Comparison of demographic characteristic to Risk preference)
**Risk preference group total**		1,034 *(62.4)*	260 *(15.7)*	183 *(11.1)*	179 *(10.8)*	1,656	
**Sex**	**Females**	585 *(68.5)*	114 *(13.4)*	83 *(9.7)*	72 *(8.4)*	854	*P* = 0.000
	**Males**	449 *(56.0)*	146 *(18.2)*	100 *(12.5)*	107 *(13.3)*	802	
**Age Group**	**<49**	19 *(52.8)*	3 *(8.4)*	7 *(19.4)*	7 *(19.4)*	36	*P* = 0.000
	**50–59**	302 *(53.0)*	106 *(18.6)*	83 *(14.5)*	79 *(13.9)*	570	
	**60–69**	383 *(63.5)*	89 *(14.8)*	70 *(11.6)*	61 *(10.1)*	603	
	**70–79**	255 *(72.4)*	49 *(13.9)*	20 *(5.7)*	28 *(8.0)*	352	
	**80+**	75 *(79.8)*	13 *(13.8)*	3 *(3.2)*	3 (3.2)	94	
	**Missing**	0 *(0.0)*	0 *(0.0)*	0 *(0.0)*	1 *(100.0)*	1	
**Mental health medication prescription**	**Yes**	183 *(67.8)*	37 *(13.7)*	23 *(8.5)*	27 *(10.0)*	270	*P* = 0.101
	**No**	851 *(61.4)*	223 *(16.1)*	160 *(11.5)*	152 *(11.0)*	1,386	
**Deprivation Measure**	**Most deprived**	139 *(71.3)*	20 *(10.3)*	18 *(9.2)*	18 *(9.2)*	195	*P* = 0.325
	**Somewhat deprived**	164 *(59.6)*	43 *(15.6)*	37 *(13.5)*	31 *(11.3)*	275	
	**Mid-deprivation**	206 *(61.9)*	57 *(17.1)*	33 *(9.9)*	37 *(11.1)*	333	
	**Not very deprived**	245 *(63.5)*	48 *(12.4)*	47 *(12.2)*	46 *(11.9)*	386	
	**Least Deprived**	280 *(60.4)*	92 *(19.8)*	47 *(10.1)*	45 *(9.7)*	464	
**Marital Status Breakdown**	**Married/Co-habiting**	694 *(62.6)*	174 *(15.7)*	119 *(10.7)*	122 *(11.0)*	1,109	*P* = 0.438
	**Single and never married**	73 *(54.9)*	26 *(19.5)*	21 *(15.8)*	13 *(9.8)*	133	
	**Separated/Divorced/Widowed**	267 *(64.5)*	60 *(14.5)*	43 *(10.4)*	44 *(10.6)*	414	
**Education level**	**None and Primary**	161 *(74.5)*	15 *(7.0)*	16 *(7.4)*	24 *(11.1)*	216	*P* = 0.035
	**Secondary**	451 *(64.5)*	96 *(13.8)*	68 *(9.7)*	84 *(12.0)*	699	
	**Tertiary**	421 *(56.9)*	149 *(20.1)*	99 *(13.4)*	71 *(9.6)*	740	
**Smoking Status**	**Never**	560 *(63.7)*	133 *(15.1)*	94 *(10.7)*	92 *(10.5)*	879	*P* = 0.619
	**Ex**	371 *(60.6)*	104 *(17.0)*	67 *(11.0)*	70 *(11.4)*	612	
	**Current**	103 *(62.4)*	23 *(14.0)*	22 *(13.3)*	17 *(10.3)*	165	
**Alcohol Consumption**	**Never**	339 *(70.2)*	67 *(13.9)*	37 *(7.6)*	40 *(8.3)*	483	*P* = 0.000
	**Occasional**	335 *(65.7)*	77 *(15.1)*	55 *(10.8)*	43 *(8.4)*	510	
	**Habitual**	360 *(54.3)*	116 *(17.5)*	91 *(13.7)*	96 *(14.5)*	663	
**BMI**	**<25**	229 (61.1)	60 (16.0)	49 (13.1)	37 (9.9)	375	*P* = 0.932
	**≥25 or <30**	461 (62.5)	116 (15.7)	76 (10.3)	84 (11.4)	737	
	**≥30**	340 (63.2)	82 (15.2)	58 (10.8)	58 (10.8)	538	

Proportional WCCs for CD8+ T, CD4+ T and CD19+ B lymphocytes, CD56+ natural killer (NK) cells, CD14+ monocytes and CD15+ granulocytes were calculated for each population group and included in the phenotype file alongside the additional covariates. Additionally, concordance plots were drawn for eight duplicate samples; average β value range; 0.001–0.99 and average r^2^ = 0.96; Supplementary Figure 1. The concordance level was not significantly different when comparing any of the demographic characteristics. Average WCCs have been included in Supplementary Table (ST) 1 for all demographic characteristics. P values have also been provided to show differences in the WCCs between the groups. All average WCC proportions were significantly different between males and females and all WCCs, except the proportion of B cells, were significantly different between the age group categories. Age, sex and WCCs were included in both models run for each of the four analyses.

Of the 1,656 individuals included in this analysis, 1,629 remained following the pre-processing of samples using RnBeads. Of these individuals, 1,020 were *risk-averse*, 255 were *mildly risk-averse*, 181 were *mildly risk-seeking* and 173 were *risk-seeking*. Four analyses were conducted, each run whilst adjusting for the minimally and maximally adjusted models in turn.

#### Analysis 1

*Assessment of single-site methylation levels in risk-averse vs. risk-seeking individuals*. The distribution of single-site DNA methylation levels was compared between the *risk-averse* group (n = 1,020) and *risk-seekin*g (n = 173) group of individuals, which passed pre-processing thresholds. *Mildly risk-adverse* and *mildly risk-seeking* individuals were excluded. In total, 46 differentially methylated CpG sites (dmCpGs) were identified between the groups where p≤x10^−5^ in the minimal model (ST2). The top-ranked dmCpG was cg09889165, p=5.39x10^−6^, which is not gene-centric.

When adjusting for all covariates, 55 dmCpGs were significantly different between the two populations (p≤x10^−5^, ST3). The top-ranked dmCpG, cg09889165, p=4.52x10^−6^, matched the results from the minimal model. Of the top-ranked dmCpGs, 41 overlap between the two analyses (ST4).

No dmCpGs were significantly different at the significance threshold of p<9x10^−8^ which has been suggested to adequately control for the false-positive rate for DNA methylation data obtained using the MethylationEPIC array [22] after adjustment for covariates.

To further assess the changes in DNA methylation between the *risk-averse* and *risk-seeking* groups, gene ontology (GO), pathway enrichment and protein interaction analyses were performed. These analyses were conducted for the genes in which the 41 overlapping top-ranked dmCpGs were located.

Enrichment based on GO terms revealed nine processes (ST5) including neuromuscular process controlling balance (p=0.07) and learning (p=0.08). Four pathways were enriched (p<0.1, ST6) including pathways in cancer (p=0.06) and aldosterone synthesis and secretion (p=0.08).

#### Analysis 2

*Assessment of single-site methylation levels in all risk-averse vs. all risk-seeking individuals*. The second analysis involved combining all individuals who passed the RnBeads pre-processing stage and were *risk-averse* (both those who were *risk-averse* and *mildly risk-averse*, n = 1,275) and all those who were *risk-seeking* (both those who were *risk-seeking* and *mildly risk-seeking*, n = 375). A total of 11 dmCpGs were identified between the groups, where p≤x10^−5^ in the minimal model (ST7). The top-ranked dmCpG was cg05768532 within *FLJ16779;NKAIN4*, p=2.64x10^−6^. No dmCpGs met the significance threshold of p<9x10^−8^ after adjustment for covariates.

When adjusting for all covariates, 11 dmCpGs were significantly different between the two populations (p≤x10^−5^, ST8) and the top-ranked dmCpG, cg05768532 within *FLJ16779;NKAIN4* (p=2x10^−6^) remained. Nine top-ranked dmCpGs overlap between the two analyses (ST9) but no processes or pathways were significantly enriched by these genes.

#### Analysis 3

*Assessment of DNA methylation in the risk-averse group compared to the remaining population*. The third analysis involved comparing the methylation patterns of the individuals from the *risk-averse* group (n = 1,020) to all the remaining individuals (n = 609) which had passed pre-processing. A total of 36 dmCpGs were identified between the groups (p≤x10^−5^) in the minimal model (ST10). The top-ranked dmCpG was cg00540637 within *C5orf45*, p=1.38x10^−6^. Three dmCpGs were located within *SMYD4* (cg09940188, p=4.88x10^−6^; cg23679982, p=8.54x10^−6^ and cg16616918, p=1.62x10^−5^).

When adjusting for all covariates, 25 dmCpGs were significantly different between the two populations (p≤x10^−5^, ST11). The top-ranked dmCpG remained (cg00540637; *C5orf45*, p=4.59x10^−6^) and the same three dmCpGs within *SMYD4* were among the top-ranked (cg09940188, p=9.64x10^−6^; cg23679982, p=1.64x10^−5^ and cg16616918, p=2.59x10^−5^). No dmCpGs met the significance threshold of p<9x10^−8^ after adjustment for covariates.

Of the top-ranked dmCpGs, 19 overlap between the two analyses (ST12). One biological process (interferon-gamma-mediated signalling pathway, p=0.041) and two molecular functions (metal ion binding, p=0.004 and double-stranded RNA binding, p=0.036) were enriched (ST13).

#### Analysis 4

*Assessment of DNA methylation in the risk-seeking group, compared to the remaining population*. The final analysis involved comparing the methylation patterns of the individuals, which passed pre-processing from the *risk-seeking* group (n = 173) to the remaining individuals (n = 1,456). A total of 116 dmCpGs were identified between the groups where p≤x10^−5^ in the minimal model (ST14). The top-ranked dmCpG was cg10811901 within *TERF2*, p=2.07x10^−6^. Three dmCpGs were located within *NWD1* (cg20249566, p=6.8x10^−5^; cg19784428, p=7.03x10^−5^ and cg19344626, p=7.69x10^−5^).

When adjusting for all covariates, 123 dmCpGs were significantly different between the two populations (p≤ x10^−5^, ST15). The top-ranked dmCpG remained (cg10811901, *TERF2*, p = 2.13x10^−6^). No dmCpGs met the significance threshold of p<9x10^−8^ after adjustment for covariates.

Of the top-ranked dmCpGs, 102 overlap between the two analyses (ST16). Enrichment analyses were undertaken for the genes in which they were located. Eleven biological processes were significantly enriched by these genes, including the negative regulation of cell proliferation (p=0.05, ST17) alongside two cellular components and four molecular functions (ST17). The mucin-type O-Glycan biosynthesis pathway was enriched (p=0.07, ST18).

## Discussion

Epigenetic alterations provide a dynamic link between genetic background and environmental exposures. These alterations have been considered to play an important role in determining an individual’s risk preference. This analysis was conducted utilising data collected as part of the NICOLA project, which assessed community dwelling older individuals from Northern Ireland.

The relationship between age and risk aversion is mixed and unclear. Some studies suggest that older individuals are more likely to be risk-averse than younger people [27]. Others have identified the opposite, whereby risk tolerance increases with age [28]. Risk-aversion has been associated with poorer decision-making in older adults [29]. The data ascertained from the NICOLA project has shown that in this Northern Irish population of older adults, 62.4% showed evidence of being risk-averse (Table 2).

Alongside age, sex and proportional WCCs, additional covariates taken into consideration in the maximal model including deprivation level, education level, marital status, smoking status, BMI, alcohol intake and whether the individual was taking any medication for mental health conditions were selected as they have been previously been linked to risk preference [8,9,30–34]. There are mixed reports regarding the relationship between education level and risk-aversion, but the majority have indicated that people with lower levels of education are more risk-seeking [35] and that for every additional year of education risk-aversion increased [33]. Those who attained higher levels of education are believed to evaluate risk more methodically [36].

The link between finance and risk preference behaviours has been assessed alongside gender and marital status. It has long been recognized that males and females approach financial risk with varying degrees of tolerance, with females being reportedly less risk-tolerant, more risk-averse [37]. The correlation of risk preference and marital status has been less consistent [37]. Some studies have suggested that married individuals are more risk-averse than those who are unmarried [38]. In 2005, one investigation proposed that married females were less risk-tolerant than those who were unmarried, and that unmarried males were more risk tolerant than married males [37].

A study including 1,094 individuals assessing risk preferences and health behaviours demonstrated significant negative correlation between risk aversion and behaviours, such as smoking, heavy alcohol intake and being overweight, alongside reluctance to wear a seat-belt [39]. Individuals who had been prescribed any nervous system drugs, psychoanaleptics, or psycholeptic drugs for mental health conditions were adjusted for within this analysis, as links have been made between several conditions and risk behaviours including attention-deficit hyperactivity disorder (ADHD), bipolar disorder, PTSD, and schizophrenia [40–42].

Top-ranked, gene-centric dmCpGs which were identified from both the minimally and maximally adjusted models when assessing the 1,656 Northern Irish participants included those within *PDLIM7, GALNT10* and *C10orf81* from Analysis 1, *FLJ16779;NKAIN4, C5orf45* and *CDCA2;KCTD9* from Analysis 2, *SMYD4, C5orf45, MLNR* and *HLA-DQA2* from Analysis 3 and *TERF2, TRAF2, PDLIM7* and *LGR5* from Analysis 4. No genes which displayed any significantly dmCpG sites have previously been linked to risk preferences, but epigenome-wide level of significance (p<9x10^−8^) was not met by any dmCpGs within these analyses whilst adjusting for covariates.

Of the genes in which top-ranked dmCpGs were located, as included within ST2-16, several have been previously linked to neural pathways, disorders and psychiatric conditions. Additionally, GO enrichment results including neuromuscular process controlling balance and learning also indicate a potential link to neural pathways. These genes include *CHD7, ADCY3, ANK3, NWD1, CNTNAP2* and *TSNAX-DISC1*, each of which has been carefully considered and contrasted as no previous link between neural disorders or psychiatric conditions, including depression, has been made for risk preference. Due to the data that we have obtained through the ontology analysis, we have chosen to focus on a potential link between neural pathways and the risk preference phenotype in this discussion.

Within *CHD7*, cg11327992 was a top-ranked dmCpG (p=5.4x10^−5^) from Analysis 3. *CHD7* is a member of the chromodomain helicase DNA-binding protein family which contributes to chromatin structure and histone variant depositions necessary to regulate gene expression. The CHD protein family is vital for neurodevelopment and variants have been linked to a range of neurological phenotypes including schizophrenia [43]. A dmCpG from Analysis 4 was located within *ADCY3*, a gene that has previously been linked to major depressive disorder [44].

*ANK3*, believed to have roles in cellular motility, proliferation and the maintenance of specialized membrane domains, contained a dmCpG reported in Analysis 3. This gene has previously been linked to schizophrenia and bipolar disorder [45]. *NWD1* (NACHT and WD Repeat Domain Containing 1) is a protein coding gene in which mutations have previously been linked to schizophrenia [46]. Several dmCpGs were identified for this gene; cg19784428 and cg19344626 from Analysis 4, and cg20249566 in both Analyses 1 and 4.

*CNTNAP2*, a gene that encodes a member of the neurexin family with functions linked to the nervous system, has been implicated in several neurodevelopmental disorders including schizophrenia, autism, ADHD and depression [47,48]. One dmCpG cg03436967 located within *CNTNAP2* was reported in Analysis 1, in both the minimally and maximally adjusted models. *LRP1* encodes a member of the low-density lipoprotein receptor family of proteins and contains one dmCpG, resulting from Analysis 4. Evidence has been accumulated which links this gene to both the maintenance of brain homoeostasis and the regulation of amyloid-β peptides in the brain and peripheral area [49].

Read-through transcription is naturally occurring between the neighbouring *TSNAX-DISC1* (translin-associated factor X and disrupted in schizophrenia 1) genes located on chromosome 1. One dmCpG, cg19999430, was reported for these genes within Analysis 4. Alterations within these genes have been linked to schizophrenia and bipolar affective disorder [50]. A SNP within this gene, rs821722, has previously been linked to addictions and opioid dependencies [51].

As indicated, several of the top-ranked genes identified in these analyses have been previously associated with or linked to neural pathways and disorders. However, this research has solely been conducted using blood-derived DNA samples due to the nature of the population-based NICOLA study and therefore there are no brain tissue samples available for assessment of gene expression levels or next-generation sequencing.

Two recent studies have been conducted which assessed methylation profiles in blood samples compared to brain tissues to ascertain whether blood samples could be used as an alternative to brain tissue. Yu et al. [52], compared methylation profiles in CD4+ lymphocytes derived from peripheral blood, with post-mortem brain tissue from 41 individuals. Using the Illumina Infinium HumanMethylation450K array, they noted significant differences in the average methylation level between the two tissue types within the same individuals.

Walton et al. [53] also compared the two tissue types and despite only including brain tissue samples from 12 individuals, they found that approximately 8% of dmCpGs showed a significant, large correlation between the two tissues and have suggested that only these dmCpGs should be considered when using blood samples to assess methylation.

An interactive tool, BECon, has been developed by Edgar et al. [54], to assist in the interpretation of blood-derived DNA methylation results as surrogates for human brain samples. The Infinium HumanMethylation450K array was used to generate the data included in this interactive tool using blood and three brain samples from 16 individuals. This has the potential for use as a validation method for dmCpGs with high levels of concordance between the tissues.

Not all dmCpGs output from our investigation of risk preference are included in the BECon interactive tool as we used the larger Infinium MethylationEPIC array for data generation. Despite this, from the top-ranked genes linked to neural pathways and across the three brain areas considered by the BECon interactive tool, a null to 0.75 positive correlation was seen for cg19344626 (*NWD1*), and a null to 0.90 positive correlation for both cg19784428 and cg20249566 (both *NWD1*) between the brain tissues and blood. A 0.50–0.75 negative correlation was noted for cg03436967 within *CNTNAP2*, whilst cg23703633 (*ANK3*), cg27449030 (*ADCY3*), and cg03668470 (*LRP1*) all showed null or negative correlations between the tissues.

When assessing the proportion of WCCs between demographic characteristics, it would be unwise to expect the proportions to be the same between different age groups and between males and females. It is expected that these will differ due to known key immunological differences between the sexes, at different stages of life [55,56]. For example, it has been shown previously that overall, males have a higher proportion of NK cells than females, but that elderly males show a more rapid decline of B and CD4+ T cells than females. Additionally, the menopause is a known contributor to the alteration of WCCs [55,56].

Risk preferences and behaviours have previously been assessed using DNA methylation techniques in a study of identical twins who were discordant for risk-taking behaviour [57]. It was established that epigenetic markers alone are unable to account for differences in their behaviour but may help to explain why one twin is able to function well, with no anxiety, in more highly dangerous situations than their other. Is it possible that our risk preferences affect lifestyles, which in turn affect epigenetic signatures, or is it the reverse? Or could it be both? As previous investigations have outlined, determining the direction of causality between risk preferences and variables such as level of education is difficult and unclear [33].

This study has some limitations. Despite reaching out to multiple colleagues, we were unable to source a complementary replication cohort with relevant phenotypic information. Additionally, only blood-derived DNA was available for analysis. It may have been advantageous to be able to assess these alterations in two independent cell samples, however previous analyses undertaken to assess methylation in blood, saliva, and/or buccal swab samples have shown high levels of correlation in the results ascertained from each [58,59]. We have employed the most comprehensive commercially available array to study DNA methylation and we have considered multiple relevant variables in our analyses, which is a particular strength of this study.

The analysis is based on cross-sectional data that were collected at one point in time, with no follow-on epigenetic or risk preference changes as yet available to study longitudinally, which is a limitation for assessing changing epigenetic profiles. It would ideally complement this cross-sectional research to be able to assess epigenetic changes connected to risk preference in this population as more samples are collected in later years. Additionally, without the benefit of longitudinal data, including the necessary phenotype information and biological samples, it is possible that any putative causal associations could be bi-directional – genetic determinants of risk preference might induce their own epigenetic signatures, while decisions made by individuals associated with their risk preferences (their diet, exercise, smoking and alcohol habits) may induce characteristic epigenetic changes. Considering such possibilities (i.e., some ‘confounders’ are really mediators) it could also be argued that our maximally adjusted models have been over-adjusted – which might also suggest that some of the p-values are overly conservative. Approaches such as Bi-Directional Mendelian Randomization may assist in the evaluation of such phenomena [60], but it has not been possible to assess this in the present study (lacking valid instruments and longitudinal follow-up). Clearly, the phenotype could be refined with follow-up information on the consequences of ‘risky’ decisions from which we might gain an improved understanding of underpinning biological mechanisms.

Potential future directions may include assessing risk preferences in conjunction with religion and religious beliefs [61] and it may also be of benefit to assess risk preferences before and after the individuals have children to monitor changes to the phenotype [32]. Finally, there may be a benefit to measuring the differences or concordance of risk preferences between parents and their children in relevant cohorts, with an attempt to apportion any correlation between shared environments and shared genes.

## Conclusion

Epigenetic research has the potential to transform the social science landscape [62]. Modifications including DNA methylation may represent important biomarkers of complementary complex genetic and environmental determinants of these traits. Several striking results from this study support further analysis of DNA methylation as an important link between measurable biomarkers and health behaviours. Data from longitudinal cohorts provide the opportunity to monitor the relationship between the two, over time.

## Supplementary Material

Supplemental Material

## Data sharing plans

NICOLA data can be accessed through DPUK and EGA.
