# Supplementary material for: An investigation into DNA methylation patterns associated with risk preference in older individuals
Source: Epigenetics. 2021 Oct 30;17(10):1159–72. doi: 10.1080/15592294.2021.1992910 (PMC9542846; doi:10.1080/15592294.2021.1992910)
Supplement: Supplemental Material [file KEPI_A_1992910_SM8449.zip › Supplementary Figure 1.docx]

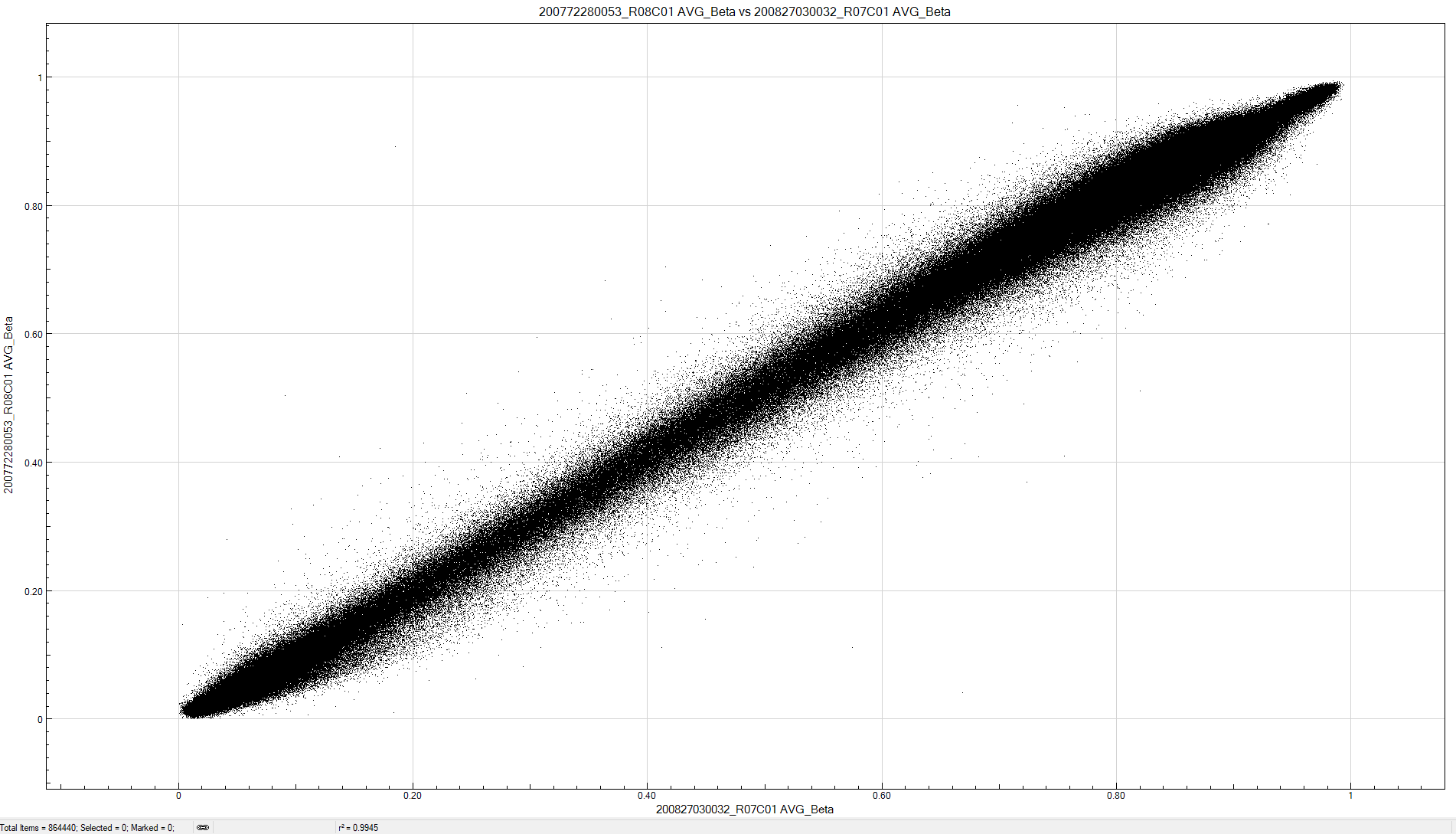


Fig. S1. Representative example concordance plot for duplicate samples.

*Representative concordance plots for a duplicate sample pair - average r^2^ for eight duplicates = 0.96.*
